# Hidden Markov model-based similarity measure (HMM-SM) for gait quality assessment of lower-limb prosthetic users using inertial sensor signals

**DOI:** 10.1186/s12984-025-01638-4

**Published:** 2025-05-12

**Authors:** Gabriel Ng, Jan Andrysek

**Affiliations:** 1 Institute of Biomedical Engineering, University of Toronto, Toronto, ON M5S 1A1 Canada; 2 Bloorview Research Institute, Holland Bloorview Kids Rehabilitation Hospital, Toronto, ON M4G 1R8 Canada

**Keywords:** Inertial sensors, Gait assessment, Gait evaluation, Gait index, Unsupervised machine learning

## Abstract

**Background:**

Gait quality indices, such as the Gillette Gait Index or Gait Profile Score (GPS), can provide clinicians with objective, straightforward measures to quantify gait pathology and monitor changes over time. However, these methods often require motion capture or stationary gait analysis systems, limiting their accessibility. Inertial sensors offer a portable, cost-effective alternative for gait analysis. This study aimed to evaluate a novel hidden Markov model-based similarity measure (HMM-SM) for assessing gait quality directly from gyroscope and accelerometer data captured by inertial sensors.

**Methods:**

Walking trials were conducted with 26 lower-limb prosthetic users and 30 able-bodied individuals, using inertial sensors placed at various lower body locations. We computed the HMM-SM score along with other established inertial sensor-based methods, including the Movement Deviation Profile, Dynamic Time Warping, IMU-based Gait Normalcy Index, and Multifeature Gait Score. Spearman correlations with the GPS, a validated measure of gait quality, were assessed, as well as correlations among the inertial sensor methods. Welch’s t-tests were used to evaluate the ability to distinguish between prosthetic subgroups.

**Results:**

The HMM-SM and other inertial sensor-based methods demonstrated moderate-to-strong correlations with the GPS (0.49 <|r|< 0.77 for significant correlations). Comparisons between different measures highlighted key similarities and differences, both in correlations and in their ability to differentiate between subgroups. Overall, the pelvis and lower leg sensors achieved significant correlations and outperformed the upper leg sensors, which did not achieve significant correlations with the GPS for any of the signal-based measures.

**Conclusion:**

Results suggest inertial sensors located at the pelvis and lower leg provide valid markers for monitoring overall gait quality, offering the potential to develop nonobtrusive, wearable systems to facilitate long-term monitoring. Such systems could enhance rehabilitation by enabling continuous gait assessment that can be easily integrated in clinical and everyday settings.

## Background

Traditional clinical practice heavily relies on observational gait analysis, despite its subjectivity and evidence indicating limitations in validity, reliability, and responsiveness [1, 2]. Instrumented gait analysis systems offer a more objective and comprehensive approach for clinicians to assess gait patterns, make informed treatment decisions, and evaluate intervention effectiveness [3, 4], ultimately improving patient outcomes [5]. Recent interest has turned to developing summarized gait quality indices as an alternative to assessing individual gait parameters. These indices provide straightforward, interpretable measures of overall gait patterns, which can be used for assessing gait problems and monitoring gait changes over time [2, 6]. Widely recognized metrics include the Gillette Gait Index (GGI) [7], Gait Deviation Index [8], and Gait Profile Score (GPS) [9], validated across various populations with gait disability. However, these indices require motion capture systems to accurately measure a full range of lower-body spatiotemporal and kinematic parameters [10]. Such systems are not readily accessible, costly and time-intensive to use, thus limiting their integration in clinical settings [3, 4]. Moreover, such systems are restricted to the lab or clinic environment, which may not be representative of real-world gait dynamics [11–13]. Development of methods tailored for wearable systems could allow for more convenient and portable solutions, facilitating their use in both clinical practice and everyday environments.

Several methods have been proposed for gait quality assessment using inertial sensors, which offer advantages such as affordability, portability, and versatility [4, 14]. The IMU-based Gait Normalcy Index (INI) [10] and Multifeature Gait Score (MGS) [15] both employ principal component analysis (PCA) on a set of gait parameters to derive a single gait quality score. The INI measures 9 parameters (3 spatiotemporal and 6 kinematic), which are normalized and transformed into orthogonal features using PCA. Deviations from a reference group of able-bodied individuals are then evaluated to determine overall gait abnormality. In contrast, the MGS defines 6 aspects of gait: amplitude, temporal, distribution, complexity, symmetry, and regularity. Each aspect incorporates various gait parameters or properties of the inertial sensor signals (e.g., skew, kurtosis, entropy). Mansour et al. then apply a PCA-driven approach to identify the most important parameters, which are compared to normative values to compute an overall gait quality score [15].

These methods still have some key drawbacks. Both algorithms require accurate and reliable measurement of many gait parameters simultaneously, which remains a significant challenge for inertial sensor systems, particularly for non-sagittal kinematics and spatiotemporal variability and symmetry parameters [16]. This may also require two or more sensors, negatively influencing the comfort and wearability of these systems [17, 18]. Furthermore, the INI and MGS rely on a predefined set of gait parameters. While some parameters such as speed are commonly regarded as important to gait quality, parameters of interest vary significantly across different populations with gait disability [19–21]. Choice of parameters may impact the performance of an overall gait quality measure when applied to different populations, so these would need to be validated in other populations. Alternatively, non-parameter-based approaches could potentially mitigate these challenges by eliminating the need for predefined, extensively validated parameter sets and circumventing the dependence on accurate parameter measurement for gait quality assessment.

Non-parameter-based approaches directly analyze the time-series signals from gait analysis systems. Barton et al. introduced the Movement Deviation Profile (MDP) [22], which evaluates deviations in time-series data using a Self-Organizing Map (SOM) trained on normative gait patterns. However, the MDP has only been validating using the pelvis, hip, knee, and ankle kinematic signals obtained from optical motion capture systems, limiting its use with inertial sensors. A robust benchmark for many machine learning tasks [23], dynamic time warping (DTW) is a distance measure for comparing time-series signals which has been used to analyze kinematics in Parkinson’s [24] and classify gait changes in lower-limb prosthetic users (LLPU) based on gyroscope signals [25]. Hidden Markov models (HMM) are another method suitable for time-series analysis, and they have been commonly used for wearable sensor gait data classification [26–28]. In our previous work, we have proposed a hidden Markov model-based similarity measure (HMM-SM) for unsupervised gait assessment using accelerometer and gyroscope data from a small set (1 to 2) of inertial sensors. The HMM-SM involves training HMMs on the inertial sensor data and measuring the similarity of these HMMs to quantify overall deviation between gait patterns. Previously, we validated that the HMM-SM could assess changes from an individual’s baseline gait patterns [29]. The current study aims to assess the performance of the HMM-SM for assessing gait normalcy and gait quality using inertial sensors in LLPU. This is accomplished through 2 main objectives. (1) Compare the HMM-SM to a validated gait quality measure to explore the clinical relevance of the HMM-SM. This study used the GPS, which is a widely cited gait quality index that has been validated in multiple populations, including LLPU [30–32]. (2) Compare other inertial sensor-based methods to the GPS to benchmark HMM-SM performance. As part of objectives 1 and 2, we also compared the scores to two functional gait measures, the prosthetic evaluation questionnaire mobility section (PEQ-MS) [33] and locomotor capabilities index (LCI-5) [34] to further explore any relationships between the gait quality methods and measures of amputee gait function. To our knowledge, this is the first study to develop and evaluate a non-parameter-based, inertial sensor gait quality assessment method with respect to a validated gait quality index such as the GPS. Additionally, no previous studies have compared inertial sensor gait quality measure with existing validated indices or compared multiple inertial sensor-based methods. Therefore, this research provides valuable evidence regarding the validity and relative performance of the HMM-SM and other methods—both parameter-based and non-parameter-based—for gait quality assessment using inertial sensor data.

## Methods

### Participants

26 LLPU completed the study protocol (23 unilateral, 3 bilateral). Individuals were included if they were above the age of 5 years and could ambulate independently on level ground. Participants included 4 types of prosthetic users: transtibial amputees (TT), transfemoral amputees (TF), Van-Nes rotationplasty (VN), and individuals with limb shortening/limb length difference (LS). 30 able-bodied participants were also recruited. Able-bodied participants were defined as individuals exhibiting no obvious gait abnormalities and having no previous history of musculoskeletal, neurological, or cardiovascular disorders. Able-bodied participants were used to form the normative group (i.e., reference) that all the gait quality measures used to calculate their scores. This is comparable to initial validations for the GGI and GPS, which used 24 and 38 participants for their reference group, respectively [7, 35]. Characteristics for the LLPU and able-bodied participants are shown in Table 1, including characteristics for each of the LLPU subgroups. Informed consent was obtained from each participant at the beginning of the data collection session. The recruitment and experimental procedure were approved by the Research Ethics Board at Holland Bloorview Kids Rehabilitation Hospital (REB-0176).

**Table 1:** Participant demographics

	Statistic	TT	TF	VN	LS	LLPU	Able-bodied
Count	n	10	7	6	3	26	30
Aids	n	1	0	0	0	1	0
Gender							
Female	n	8	4	4	0	16	21
Male	n	2	3	2	3	10	9
Age (years)	Mean (σ)	29.9 (18.5)	34.6 (13.8)	16.0 (6.2)	20.3 (13.3)	26.8 (15.6)	25.2 (6.9)
Height (m)	Mean (σ)	1.63 (0.12)	1.69 (0.12)	1.57 (0.15)	1.44 (0.24)	1.61 (0.15)	1.70 (0.08)
Weight (kg)	Mean (σ)	73.8 (21.9)	63.4 (14.2)	55.5 (16.1)	38 (18.5)	62.7 (20.9)	66.2 (10.6)
Time (years)	Mean (σ)	16.1 (13.9)	12.8 (11.6)	4.3 (4.8)	19.3 (14.6)	12.8 (12.3)	N/A

Aids, participants using walking aids (e.g., cane, walker, etc.); Time, time since amputation (same as age if congenital); σ, standard deviation

*TT* transtibial amputee, *TF* transfemoral amputee, *VN* Van Nes rotationplasty, *LS* limb shortening, also known as limb length difference, *LLPU* lower-limb prosthetic user

### Data acquisition

**Fig. 1:**
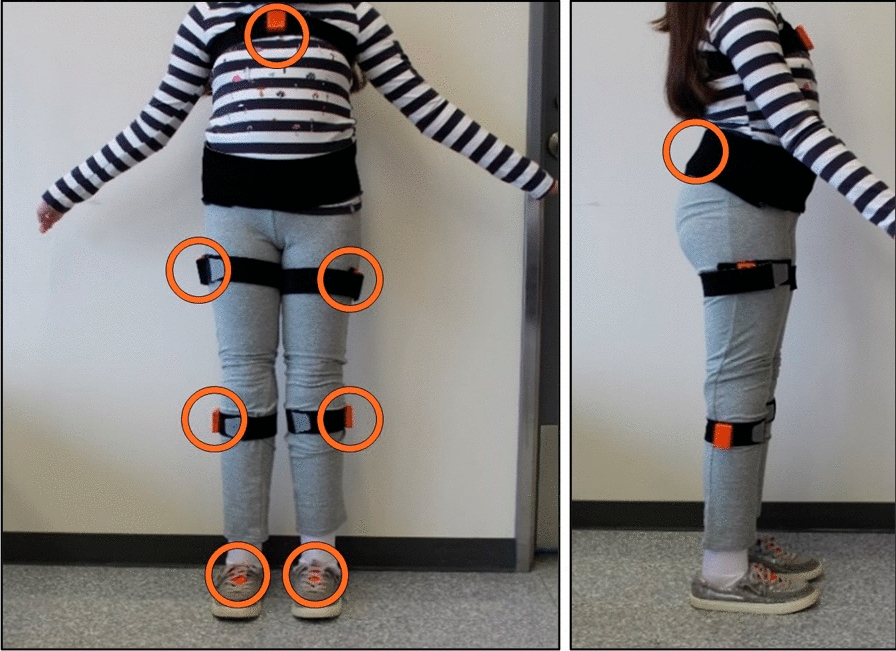
Example participant setup for the Xsens Awinda. Able-bodied and LLPU participants followed the same setup. Sensors on bridge of both feet, lower legs below the knee (shank), outside of upper legs parallel with sagittal plane (thigh), center of pelvis on the sacrum, and center of sternum. Free acceleration and angular velocity were used to train the ML models and calculate signal parameters

Participants were instrumented with the Xsens Awinda system (Xsens Technologies BV, Enschede, Netherlands) and wore 8 inertial sensors located on the lower body and sternum, as shown in Fig. 1. This system has been well-validated for kinematic measurements, demonstrating excellent reliability in the sagittal plane and fair-to-excellent reliability in the other planes [36]. The placement of the sensors followed anatomical markers suggested in the Xsens user manual [37]. The Xsens collected orientation-free accelerometer (range ± 16 g) and angular velocity data (range ± 35 rad/s) as well as foot contacts, 3D position and orientation, and kinematic signals at 100 Hz. The accelerometer and angular velocity signals were down-sampled to 40 Hz and used by the signal-based ML models as well as to calculate any signal parameters (e.g., skew, kurtosis), while the foot contact, position, orientation, and kinematics were used to calculate spatiotemporal and kinematic gait parameters.

All participants completed walking trials along a 15 m straight pathway, stopping and turning around at either end. The first and last gait cycles of each pass were excluded to ignore the starting and stopping portion. LLPU participants completed 20 passes, which was able to capture at least 100 steady-state gait cycles for each participant. 100 gait cycles were sampled randomly from each of the LLPU participants for calculating the gait quality scores. Able-bodied participants were instructed to complete 10 passes, which resulted in at least 50 gait cycles. We randomly sampled 10 gait cycles from each able-bodied participant and aggregated these to form the normative/reference group used for each of the gait quality measures, similarly to the process by Wang et al. [10].

LLPU participants also completed 2 self-report functional measures, the Prosthesis Evaluation Questionnaire-Mobility Section (PEQ-MS) and the Locomotor Capabilities Index (LCI-5). These were included to assess relationships between gait mobility capacity as measured by the questionnaires and outputs from the gait quality scores, and to potentially provide more information concerning the clinical relevance of the inertial sensor methods.

### Gait quality measures

Multiple gait quality measures were used for this study. All methods were able to provide a continuous output of gait similarity or distance (as opposed to a discrete classification scheme), analogous to the previously validated gait indices such as the GPS, GDI, and GGI. The primary inertial sensor-based method used was the HMM-SM, which was compared against 4 other inertial sensor-based measures. Detailed descriptions for these methods are provided in the subsequent sections. We categorize inertial sensor-based methods into two types: signal-based and parameter-based. Signal-based methods utilize raw time-series data from wearable sensors (such as gyroscopes and accelerometers) directly as inputs to the model for determining gait quality scores, and includes the HMM-SM, MDP, and DTW. In contrast, parameter-based methods involve calculating discrete parameters which can be derived from inertial sensor data, which are then used to assess overall gait quality, and include the INI and MGS.

For this study, the GPS was used as the gold standard gait quality measure to evaluate the performance of the inertial sensor gait quality methods. It was calculated as outlined in literature [35] using kinematic data obtained from the Xsens. Specifically, the GPS takes into account the pelvic orientation, foot progression, and joint angles from the hips, knees, and ankles. A Euclidean distance is calculated throughout the gait cycle between a participant’s gait and a set of normative gait (e.g., able-bodied individuals) to determine a gait deviation score. To calculate an individual’s GPS score, we used the mean score of the 100 sampled gait cycles.

All the gait quality measures in this study, including the GPS, derive their score by comparing LLPU participant data to a normative dataset representing ideal gait, typically able-bodied gait. The normative (i.e., reference) dataset in this study was formed as detailed in the previous section (Data acquisition) using the 10 randomly sampled gait cycles from each able-bodied participant.

### Hidden Markov model similarity measure (HMM-SM)

The HMM-SM leverages a computationally low-cost method developed by Sahraeian and Yoon [38] to assess the similarity of HMM models. Each HMM $\lambda$ is characterized by 2 main matrices *A* and *B*. *A* is an *N* × *N* matrix where each element ${a}_{i, j}$ represents the probability the system would transition from state *i* to state *j* at a given time. The number of states, *N*, is a hyperparameter of the system. *B* is the emissions probability matrix consisting of *N* elements ${b}_{1},\dots ,{b}_{N}$ where each element represents the likelihood of system outputs for a given state. These likelihoods are represented by a $\mu$ (mean) and covariance matrix. To quantify the similarity of two HMMs (${\lambda }_{a}\text{ and }{\lambda }_{b}$), we first calculate a state-correspondence matrix *Q* where each element ${q}_{i,j}$ indicates the overall similarity between the emission matrix elements ${b}_{a}[i] \text{and }{b}_{b}[j]$, evaluated using symmetric Kullback–Leibler divergence. Subsequently, a similarity measure $S({\lambda }_{a}||{\lambda }_{b})$ is calculated based on the sparsity of *Q:*

1 $$S({\lambda }_{a}|\left|{\lambda }_{b}\right)\triangleq \frac{1}{2}\left[\frac{1}{M}\sum_{i=1}^{M}H\left({r}_{i}\right)+\frac{1}{{M}{\prime}}\sum_{i=1}^{{M}{\prime}}H\left({c}_{j}\right)\right]$$

where *r*_*i*_ is the *i-*th row of *Q*, *c*_*j*_ is the *j-*th column of *Q*, and *M* and ${M}{\prime}$ are the number of rows and columns in *Q*. $H({\varvec{u}})$ represents the normalized Gini Index which returns the sparsity of vector ***u*** from 0 to 1. A similarity score, $S({\lambda }_{a}||{\lambda }_{b})$, closer to 1 indicates a higher degree of similarity between the HMMs. Using these properties, we can compare individual’s gait to that of ideal or normative gait to calculate a single gait quality score, where greater similarity to normative gait represents higher gait quality.

We conducted preliminary validation simulating gait perturbations in able-bodied individuals, which provided insights into hyperparameters and sensor configurations to use for the HMM-SM [29]. For this study, 5-state HMMs were used. Experiments demonstrated including multiple gait cycles increased HMM training consistency. This is also done for other HMM-based methods such as in Cuzzolin et al. which uses gait data from a 10 m pass to train their HMM models [26]. For each participant, we transformed their randomly sampled set of 100 gait cycles into a set of multi-cycle sequences. This was accomplished by using a sliding window to iteratively select groups of 10 gait cycles, which were then concatenated along the time axis. The concatenated sequences were used to train the HMM model for each participant (${\lambda }_{p}$). Each HMM, ${\lambda }_{p}$, was then compared to the HMM trained on the normative dataset (${\lambda }_{control}$) using the similarity measure from Eq. 1 to determine the participant’s gait quality score. An overview of this is provided in Table 2, along with the other inertial sensor-based gait quality measures.

**Table 2:** Summary of the inertial sensor-based gait quality measures

Gait measure	Gait features used	Data analysis and score calculation
INI *(Parameter-based)*	9 gait parameters: 3 spatiotemporal (GD, SL, and PSP) and 6 kinematic (MV, MH, MHD, MAB, MAD, and SRM)	- Determine eigenvalues and eigenvectors based on normative gait using PCA - Transform individual and normative gait parameters into new PCA-derived coordinate system - Euclidean norm used to calculate distance between normative and individual gait (i.e., overall deviation)
MGS *(Parameter-based)*	6 aspects of gait (amplitude, temporal distribution, complexity, symmetry, and regularity), each comprised of a mix of spatiotemporal or signal-based (e.g., skew, kurtosis) gait parameters	- Calculate eigenvalue/eigenvector pairs, keeping those with eigenvalue ≥ 1 - Per each remaining eigenvector, determine correlation of gait parameters. Keep gait parameter from each “aspect” with the highest correlation - Using reduced parameter set, calculate z-scores of each parameter based on mean and standard deviation of the normative gait set. Standardize these between 0 and 1 - Using standardized deviation scores per parameter, calculate mean “aspect” scores as well as the overall mean deviation
HMM-SM *(Signal-based)*	Triaxial accelerometer and gyroscope signals from lower-body inertial sensors	- Each participant’s gait data transformed into multi-gait cycle sequences, using a sliding window to iteratively select groups of 10 gait cycles which are subsequently concatenated along the time axis. Similar to that used in our previous work [29] - Do same for normative dataset (able-bodied gait) - Train HMM on the normative able-bodied dataset (${\lambda }_{control}$) - Train HMM on the participant’s transformed dataset (${\lambda }_{p}$) - Compute similarity between participant and normative HMMs,$S({\lambda }_{p}|\left|{\lambda }_{control}\right)$
MDP *(Signal-based)*	Triaxial accelerometer and gyroscope signals from lower-body inertial sensors	- Train self-organizing map (SOM) on the normative data - For each time point in gait cycle, find best-matching unit in self-organizing map based off Euclidean norm distance - Overall score equals mean distance across the gait cycle
DTW *(Signal-based)*	Triaxial accelerometer and gyroscope signals from lower-body inertial sensors	- Compute distance between each participant gait cycle and able-bodied gait cycle using tslearn algorithm for multivariate time series [39] - Determine the mean distance for all the comparisons to determine overall DTW-based score

Includes features used by each method and a summary of how the gait quality score is calculated. Measures split into parameter-based (top) and signal-based measures (bottom)

*INI* IMU-based Gait Normalcy Index, *MGS* Multifeature Gait Score, *HMM-SM* hidden Markov model-based similarity measure, *MDP* Movement deviation profile, *DTW* Dynamic time warping, *GD* gait cycle duration, *SL* stride length, *PSP* percentage swing phase, *MV* maximum ankle velocity, *MH* maximum ankle height, *MHD* ankle horizontal displacement at MH, *MAB* maximum ankle abduction, *MAD* maximum ankle adduction, *SRM* shank range of motion in swing phase, *PCA* principal component analysis

For this study, we tested three sensor configurations identified from preliminary investigations: pelvis, combined upper leg signals, and combined lower leg signals [29]. The single-sensor data were 6 × T arrays (tri-axial gyroscope and accelerometer signals, over T time points following the concatenation process). This meant for the pelvis configuration, the HMMs were trained on 6 × T arrays, whereas for the combined sensor configurations (upper leg and lower leg), the sensor data was stacked such that the HMMs were trained on 12 × T arrays.

### Other inertial sensor gait quality measures

In addition to our method, the HMM-SM, we calculated scores for the INI, MGS, and MDP to serve as benchmarks against which to compare the HMM-SM’s performance. All methods were calculated as outlined in literature [10, 15, 22]. As an additional benchmark, we also evaluated DTW, which is a common baseline for evaluating time-series model performance [23]. For this work, we computed an average DTW distance between LLPU and able-bodied gait cycles. This was done using the tslearn library for determining the DTW distance of multivariate time series [39]. Table 2 provides a summary of the processes for the inertial sensor-based methods.

Neither the INI nor MGS publications included whether the right or left side were used for calculating the non-symmetry parameters, so gait parameters from the prosthetic side were used for unilateral prosthetic users, while the averages of right and left were used for bilateral prosthetic users. For the signal-based measures, the MDP and DTW, we tested the same sensor configurations as for the HMM-SM (pelvis, upper legs, and lower legs). For the MDP, instead of using the different kinematic signals as demonstrated by Barton et al. [22], the SOM was trained using the tri-axial accelerometer and gyroscope signals. To our knowledge, the MDP has yet to be evaluated using just wearable sensor signals. To calculate the INI, MGS, and MDP scores for each participant, we averaged the scores of the 100 sampled gait cycles, the same as for the GPS.

### Data analysis

The Spearman’s rank correlation coefficient was used to evaluate the relationship between the various gait quality measures used in the study. The primary correlations of interest were between the GPS and the HMM-SM from the 3 sensor locations as well as between the GPS and other inertial sensor-based gait quality measures. These were used to evaluate concurrent validity with respect to the GPS and benchmark the performance of the HMM-SM.

Because of how the scores are constructed, we would expect the HMM-SM to have a negative correlation with the GPS (i.e., r-value < 0). This is due to the HMM-SM being a similarity measure between 0 and 1, where a score of 1 indicates no deviation, and decreasing scores (closer to 0) indicate greater deviation (i.e., lower similarity) with respect to the reference dataset. This is opposite to the GPS, whose range starts at 0 (no deviation) and in which increasing scores correspond to greater deviation with respect to the reference gait. Like the GPS, the remaining inertial sensor-based measures (MDP, DTW, INI, and MGS) should anticipate positive correlations with the GPS, as they are all formulated such that a score of 0 indicates no deviation, and increasing scores correspond to greater deviation from reference gait.

We also calculated the correlation coefficients between the different inertial sensor-based measures to identify any significant relationships. Lastly, we assessed correlations between the inertial sensor-based methods and the self-report measures. For this study, we considered an absolute value of r less than 0.1 as negligible correlation, 0.1–0.4 as weak, 0.4–0.7 as moderate, 0.7–0.9 as strong, and greater than 0.9 as very strong [40]. An alpha value of 0.05 was used to determine significance for all the statistical tests. To mitigate the risks arising from multiple comparisons, we applied a Benjamini-Hochberg (BH) correction with a false discovery rate (*q*) of 0.05 to determine significance of the correlations [41]. We used the protocol described by Benjamini and Hochberg [42] to calculate adjusted *p*-values which we report in this paper.

We also aimed to evaluate the discriminative validity by assessing differences in the gait quality measures between the different LLPU subgroups (TT, TF, VN, and LS). A Shapiro–Wilk test was used to evaluate normality of the data within each subgroup. A repeated measures ANOVA (RM-ANOVA) was used to determine whether any differences were present between the different subgroups for each of the inertial sensor-based algorithms. Post-hoc Welch’s t-tests were then performed to identify which groups significantly differed (alpha = 0.05).

## Results

### Correlations between gait quality measures

**Fig. 2:**
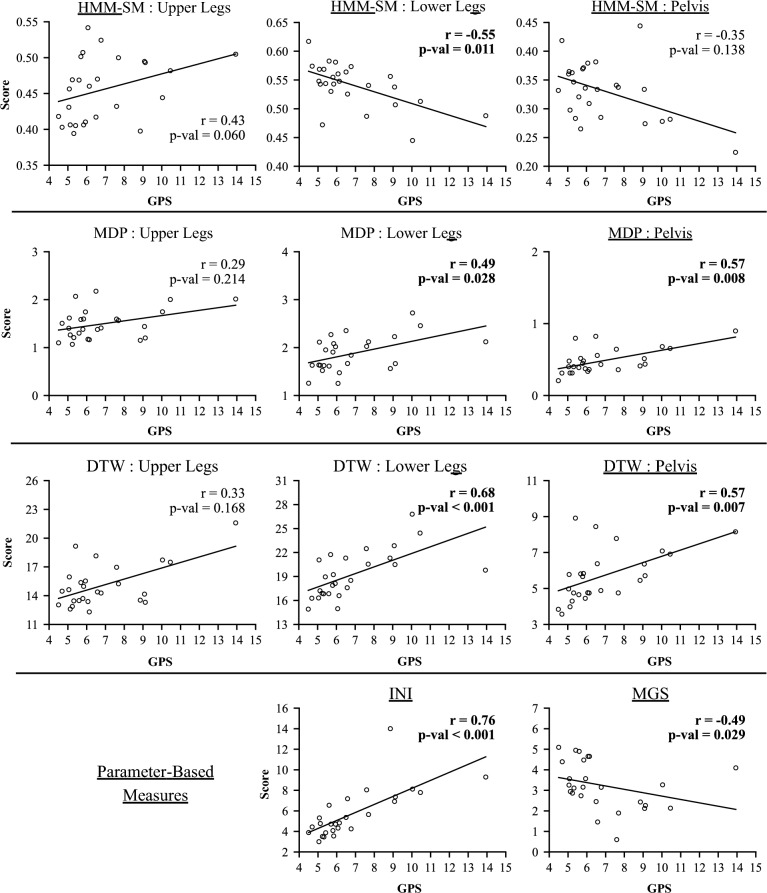
Correlations between the GPS (horizontal axis) and inertial sensor-based measure scores (vertical axis). The HMM-SM, DTW, and MDP algorithms are grouped, with plots for each of the sensor configurations evaluated in the study (upper leg sensors, lower leg sensors, and pelvis sensor). Plots with bolded correlation and adjusted *p*-values indicate significant correlation with the GPS (α = 0.05) for that configuration. R-values and *p*-values obtained using Spearman’s rank correlation coefficient as outlined in the methodology

We analyzed eleven scores derived from inertial sensor gait quality measures in relation to the GPS. This includes three sensor configurations for the HMM-SM, MDP, and DTW, and one score each for the INI and MGS. Figure 2 displays the comparison of inertial sensor scores with GPS results, and Table 3 summarizes the Spearman’s rank correlation tests comparing all the different gait quality measures. Because the HMM-SM is a similarity measure, higher scores correspond to better gait quality (i.e., greater similarity to reference dataset). In contrast, higher scores for the other measures (INI, MGS, MDP, DTW, and GPS) should reflect worse gait quality as it indicates increased gait deviation. The results showed many significant correlations between the inertial sensor-based measures and the GPS. Additionally, there were many correlations between the different sensor configurations and between the different inertial sensor-based methods.

**Table 3:** Correlations across gait quality measures

	GPS	HMM-SM upper	HMM-SM lower	HMM-SM pelvis	MDP upper	MDP lower	MDP pelvis	DTW upper	DTW lower	DTW pelvis	INI
HMM-SM upper	0.428 (0.06)										
HMM-SM lower	−0.549* (0.011)	−0.294 (0.213)									
HMM-SM pelvis	−0.350 (0.138)	−0.339 (0.153)	0.412 (0.07)								
MDP upper	0.290 (0.214)	−0.031 (0.908)	−0.167 (0.471)	−0.197 (0.413)							
MDP lower	0.491* (0.028)	0.188 (0.43)	−0.411 (0.07)	−0.285 (0.218)	0.860* (<0.001)						
MDP pelvis	0.567* (0.008)	0.145 (0.52)	−0.470* (0.036)	−0.319 (0.181)	0.720* (<0.001)	0.748* (<0.001)					
DTW upper	0.328 (0.168)	−0.018 (0.944)	−0.219 (0.358)	−0.147 (0.52)	0.947* (<0.001)	0.815* (<0.001)	0.757* (<0.001)				
DTW lower	0.677* (<0.001)	0.147 (0.52)	−0.535* (0.014)	−0.256 (0.278)	0.594* (0.005)	0.832* (<0.001)	0.730* (<0.001)	0.611* (0.003)			
DTW pelvis	0.575* (0.007)	0.167 (0.471)	−0.518* (0.018)	−0.239 (0.316)	0.677* (<0.001)	0.705* (<0.001)	0.968* (<0.001)	0.733* (<0.001)	0.718* (<0.001)		
INI	0.761* (<0.001)	0.196 (0.413)	−0.415 (0.07)	−0.296 (0.213)	0.234 (0.323)	0.392 (0.087)	0.443 (0.052)	0.295 (0.213)	0.645* (0.002)	0.432 (0.059)	
MGS	−0.487 (0.029)	−0.185 (0.431)	0.533* (0.014)	0.007 (0.972)	−0.039 (0.889)	−0.389 (0.088)	−0.286 (0.218)	−0.097 (0.679)	−0.578* (0.007)	−0.306 (0.202)	−0.449* (0.049)

R-values listed for each comparison, with corresponding adjusted *p*-values in parentheses. Correlations with achieved significance threshold (alpha = 0.05) are bolded and have an asterisk (*). The first column shows results comparing the inertial sensor-based measures to the GPS, while the rest of the cells indicate comparisons between the different inertial sensor-based measures. Of note, the MGS-GPS comparison is not highlighted, as the correlation was significant but in the incorrect direction as expected

*GPS* Gait Profile Score, *HMM-SM* hidden Markov model-based similarity measure, *MDP* Movement deviation profile, *DTW* Dynamic time warping, *INI* IMU-based Gait Normalcy Index, *MGS* Multifeature Gait Score

All the signal-based measures had at least one sensor configuration which was significantly correlated with the GPS, and these were all moderate correlations. The HMM-SM lower leg configuration showed a moderate negative correlation with the GPS (r = −0.55, *p* = 0.011). Other time-series measures which were significantly correlated with the GPS were the DTW lower leg (r = 0.68, *p* < 0.001), DTW pelvis (r = 0.57, *p* = 0.007), MDP lower leg (r = 0.49, *p* = 0.028), and MDP pelvis (r = 0.57, *p* = 0.008). HMM-SM and MDP exhibited comparable performance, with moderate correlations with the GPS for the lower leg configurations. Among the signal-based measures, DTW demonstrated the strongest correlation with the GPS, with the lower leg configuration results approaching a strong correlation. In general, across the signal-based measures (HMM-SM, MDP, and DTW), the sensor location on the upper legs performed worse than the pelvis and the lower limbs.

The INI demonstrated the strongest correlation with the GPS (r = 0.76, *p* < 0.001), while the MGS showed a moderate negative correlation (r = −0.49, *p* = 0.029). Notably for the MGS, the negative correlation was opposite what would be expected, indicating MGS scores improved as gait kinematics deviated from the reference dataset. This was the only measure to report significant correlations in the opposite direction as expected.

There were also many correlations among the different inertial sensor configurations, as seen in the Table 3 non-GPS columns. Comparing different sensor configurations with a given algorithm (e.g., DTW upper leg vs. DTW pelvis), the MDP exhibited strong correlations between the sensor configurations, while DTW exhibited moderate correlation between upper and lower leg (r = 0.61) and strong correlations between the pelvis and both the upper and lower leg configurations (r = 0.73 and r = 0.72, respectively). The HMM-SM sensor configurations did not show significant correlations with each other.

Comparing different measures, the HMM-SM lower leg configuration showed moderate correlation with the MDP pelvis, DTW lower leg and pelvis, INI, and the MGS (HMM-SM Lower column, Table 3). The MDP and DTW measures were highly correlated, with significant correlations observed for all the MDP and DTW comparisons. Only the comparison between MDP-Upper and DTW-Lower was not strong (r = 0.59), and the MDP-DTW upper and MDP-DTW pelvis correlations were both very strong (r > 0.90). The DTW lower leg configuration also showed moderate correlations with both the parameter-based measures (DTW Lower column, Table 3). The INI and MGS had a significant negative correlation.

Lastly, we compared the inertial sensor-based scores to the results from the functional measure questionnaires – the PEQ-MS and LCI-5 – to identify any relationships between the measures and assess whether the inertial sensor-based measures could be used to estimate functional ability in LLPU. As shown in Table 4, neither functional measure demonstrated significant correlations with any of the gait quality measures.

**Table 4:** Correlations with functional measures

	GPS	HMM-SM upper	HMM-SM lower	HMM-SM pelvis	MDP upper	MDP lower	MDP pelvis	DTW upper	DTW lower	DTW pelvis	INI	MGS
PEQ-MS	0.106 (0.934)	0.051 (0.934)	0.081 (0.934)	0.08 (0.934)	0.161 (0.934)	0.126 (0.934)	0.177 (0.934)	0.177 (0.934)	0.274 (0.934)	0.123 (0.934)	0.027 (0.943)	0.113 (0.934)
LCI-5	−0.081 (0.934)	0.047 (0.934)	0.075 (0.934)	0.23 (0.934)	−0.066 (0.934)	−0.058 (0.934)	−0.172 (0.934)	−0.066 (0.934)	−0.04 (0.934)	−0.146 (0.934)	−0.45 (0.746)	−0.009 (0.967)

R-values provided with corresponding adjusted *p*-values in parentheses. Correlations with achieved significance threshold (alpha = 0.05) are bolded. None of the assessed correlations to functional measures were statistically significant in this study

*GPS* Gait Profile Score, *HMM-SM* hidden Markov model-based similarity measure, *MDP* Movement deviation profile, *DTW* Dynamic time warping, *INI IMU*-based Gait Normalcy Index, *MGS* Multifeature Gait Score, *PEQ-MS* Prosthetic Evaluation Questionnaire – Mobility Section, *LCI-5* Locomotor Capabilities Index-5

### Differences between prosthetic levels

**Fig. 3:**
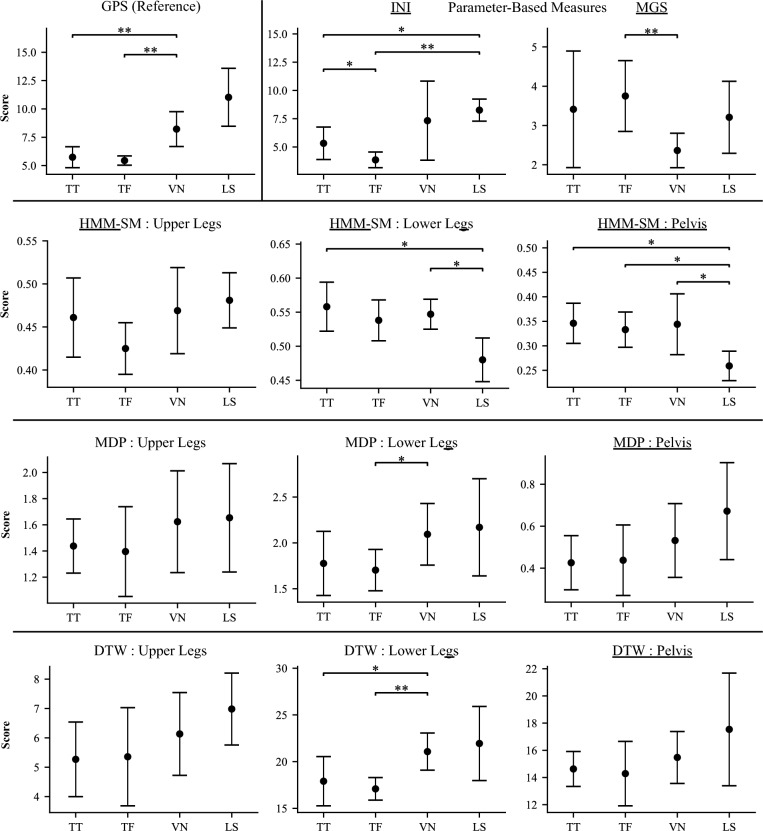
Results from post-hoc t-tests comparing the different amputee levels. Significance bars indicate levels where statistical significance was detected, with corresponding significance level (*≤0.05, **≤0.01, and ***≤0.001). The GPS (reference measure) results are shown in the top left. The VN group scores were significantly higher than TT or TF amputees. Differences between subgroups were also found in the following inertial sensor configurations: INI, MGS, HMM-SM Lower Leg, HMM-SM Pelvis, MDP Lower Leg, and DTW Lower Leg. *TT* Transtibial, *TF* Transfemoral, *VN* Van Nes, *LS* Limb Shortening

We evaluated differences among prosthetic levels by analyzing gait quality measures across different prosthetic user types (TT, TF, VN, and LS). Mean and standard deviation values for each subgroup are presented in Fig. 3. Welch’s t-tests were used to identify significant differences. The results from the Welch’s test are presented in Table 5 and demonstrate that the gait quality measures varied in which subgroups they differentiated between.

**Table 5:** Comparison of prosthetic levels

Algorithm	TT–TF	TT–VN	TT–LS	TF–VN	TF–LS	VN–LS	Differences per configuration
GPS	0.360	**0.008**	0.065	**0.006**	0.062	0.187	2
HMM-SM upper	0.067	0.747	0.441	0.093	0.068	0.692	0
HMM-SM lower	0.235	0.472	**0.025**	0.529	0.060	**0.046**	2
HMM-SM pelvis	0.496	0.942	**0.012**	0.712	**0.022**	**0.027**	3
MDP upper	0.780	0.316	0.465	0.292	0.409	0.923	0
MDP lower	0.611	0.098	0.327	**0.040**	0.263	0.837	1
MDP pelvis	0.875	0.233	0.200	0.349	0.214	0.422	0
DTW upper	0.736	0.365	0.347	0.339	0.305	0.484	0
DTW lower	0.447	**0.016**	0.192	**0.003**	0.153	0.743	2
DTW pelvis	0.908	0.246	0.114	0.383	0.143	0.397	0
INI	**0.014**	0.229	**0.010**	0.059	**0.006**	0.565	3
MGS	0.569	0.060	0.783	**0.006**	0.440	0.246	1
Differences per Subgroup	1	2	3	4	2	2	

Results from post hoc Welch’s t-test comparing the different prosthetic levels. *p*-values for configurations which were statistically significant are bolded and underlined. X–Y indicates the two levels being compared. Differences per Configuration indicates the number of differences identified by each algorithm/sensor configuration (count of significant results in each row). Differences per Subgroup indicates number of algorithms that showed significant difference for each of the subgroup comparisons (count of significant results in each column)

*TT *Transtibial, *TF *Transfemoral, *VN *Van Nes, *LS *Limb Shortening

The GPS detected 2 subgroup differences. Based on the GPS the VN group had significantly worse gait quality compared to both the TT (*p* = 0.008) and TF (*p* = 0.006) groups, as evidenced by higher scores. The mean GPS score for the LS group was 5.5° higher than those of the TT or TF groups, well above the minimal clinically important difference of 1.7° in LLPU identified by Carse et al. [30]. However, the differences between the TT-LS and TF-LS groups were borderline significant (*p* = 0.065 and *p* = 0.062, respectively), but did not reach the significance threshold of 0.05, potentially due to the smaller sample size of the LS group (n = 3).

All measures detected differences between at least one set of subgroups. The HMM-SM captured a total of 5 significant sub-group differences, across 2 sensor locations, which was the most among the signal-based measures. Specifically, for the HMM-SM lower leg configuration, LS participants scored significantly lower than the TT and VN groups, while for the pelvis configuration, LS participants scored lower than all three other prosthetic types. The DTW lower leg configuration best matched the GPS as it showed the same 2 significant differences as the GPS, between the LS group and both the TT and TF participants. The other two locations showed no differences. The MDP only revealed one significant sub-group difference in total across all three sensor locations, between TF and VN. In general, across the signal-based measures, the upper legs configuration performed worse than the pelvis and lower legs, as none of the HMM-SM, DTW, or MDP upper leg configurations revealed any significant differences between the prosthetic types. The INI detected 3 differences, including between the LS group and both the TT and TF groups, as well as between the TT and TF groups. The MGS revealed a single difference, between TF and VN.

There were no clear patterns for which subgroups the measures discriminated, as these varied between measures and sensor locations. For example, the HMM-SM only detected differences between LS and the other subgroups, while DTW results differentiated between TF and VN. TF-VN difference was most commonly identified (4 instances), followed by 3 for TT-LS, 1 for TT-TF, 2 for the remaining subgroups.

We also explored partitioning the LLPU into only two groups. To do this, we considered the VN and the TT as one group and the LS and TF as the second group. For instance, some studies consider VN also as below knee amputees in their analysis [43]. However, in this configuration, there were no statistically significant differences between the two groups for any of the gait quality measures.

## Discussion

This study aimed to evaluate the proposed HMM-SM as a novel method for assessing gait quality using inertial sensor signals. To accomplish this, we examined the concurrent validity of the HMM-SM comparing its results with those obtained from the GPS, which were derived from the same walking trials. We also compared the HMM-SM and GPS with other inertial signal-based gait quality measures and time-series analysis techniques from the literature to benchmark its performance against existing methods. Additionally, we investigated the discriminative capabilities of these methods by analyzing how they differentiated between different types of prosthetic users. At a high level, all of the signal-based measures including the HMM-SM were at least moderately correlated with the GPS and were able to discriminate between at least 2 LLPU subgroups. Hence, these findings provide both confirmatory and new evidence (particularly in the case of HMM-SM) that inertial sensor signals could be used to assess overall gait quality. A summary of advantages and disadvantages of the inertial sensor-based algorithms, based on findings from the study, is included in Table 6 (excluding the MGS), with in depth discussion in the following sections.

**Table 6:** Summary of characteristics for inertial sensor-based gait quality measures

Algorithm	Potential advantages	Potential disadvantages
HMM-SM	- Could allow for additional analysis of spatiotemporal gait features - Particularly effective at discriminating compensatory strategies employed by limb-difference (e.g., at the pelvis)	- Inconsistent performance depending on sensors used
MDP	- Can form deviation curve throughout the gait cycle, as done in [22] - Similar performance using different sensors - Only need to iterate over control data once, when training the SOM	- Very similar performance as DTW with added algorithmic complexity
DTW	- Closely approximates GPS performance - Similar performance using different sensors - Simple to calculate	- Computationally expensive for larger datasets
INI	- Closely approximates GPS performance	- Requires accurate estimation of multiple kinematic and spatiotemporal parameters - No inclusion of symmetry parameters

### Inertial sensor-based measures for gait quality assessment

Among the signal-based methods, DTW exhibited the strongest correlations with the GPS, suggesting that even a relatively simple time-series analysis method like DTW could be used to evaluate overall gait quality. Studies in other domains such as time-series classification demonstrate that even 1-Nearest Neighbor DTW is a robust benchmark, with state-of-the-art algorithms failing to surpass it or achieving only marginal accuracy improvements [23, 44]. However, the HMM-SM may offer additional advantages over DTW beyond correlation with the GPS. For example, previous studies have used HMMs for gait event identification and gait phase analysis [45–47]. Therefore, the HMM-SM framework could be used to assess overall gait quality while simultaneously allowing for extraction of gait events and parameters which may be clinically useful.

Results suggest that optimal locations for assessing gait patterns using inertial sensors are the lower legs and pelvis. DTW and MDP both demonstrated significant correlations with the GPS for lower leg and pelvis configurations. The HMM-SM only showed significant correlations for the lower-leg, but this is in line with findings from initial investigations with able-bodied individuals that showed decreased HMM-SM reliability using the pelvis compared to both the upper leg and lower leg configurations [29]. None of the signal-based algorithms (HMM-SM, MDP, and DTW) showed significant correlation with the GPS using upper leg sensors. Although sensor placement and quantity vary across gait studies, results from this study support previous literature which indicates that the most common locations are the pelvis and lower legs [48–50].

Comparing GPS correlations between the signal-based and parameter-based measures, the HMM-SM and other signal-based measures (MDP and DTW) outperformed the MGS, whose scores improved as GPS scores worsened. In contrast, the INI showed the strongest correlation among the inertial sensor-based measures (r = 0.76, *p* < 0.001), indicating it could be a valid measure of gait quality. This was notable given that the INI uses a different set of gait parameters than the GPS, suggesting that a PCA-based approach can effectively quantify overall gait deviation. However, the MGS, which also used a PCA-based approach, decreased as the GPS and INI scores worsened. This discrepancy highlights how the choice of parameters and techniques can significantly impact the performance of parameter-based gait quality measures. Variations in parameter importance across different disability populations could contribute to this. Wang et al. suggest that parameters for a gait quality measure may need adjustments for different populations, particularly since symmetry measures, which are crucial for hemiplegic or asymmetric gait, were not included in the INI [10].

The HMM-SM, DTW, and MDP methods, which do not require extensive gait parameter validation and tuning, could be more adaptable across clinical populations. This would also reduce the reliance on accurately and reliably measuring a broad set of gait parameters. This is advantageous given that wearable systems can be less accurate than non-wearable systems, require complex algorithms for parameter assessment (e.g., obtaining the motion trajectory and kinematics for the INI), and typically assess a limited number of gait parameters [1, 3].

### Correlations between inertial-sensor based methods

None of the HMM-SM sensor configurations showed significant correlations with each other. We might have expected some correlations given that movements in the pelvis, upper legs, and lower legs might impact each other. Baker et al. observed in their original validation of the GPS that individual component scores for different kinematics did not strongly correlate with each other [35]. This suggests that changes in gait patterns may have varied effects across different lower body locations and movement planes, potentially explaining the lack of significant correlations among HMM-SM configurations.

In contrast, comparing the sensor configurations within each of the DTW and MDP results, the three configurations were highly correlated with each other. The DTW lower leg versus upper leg exhibited a moderate correlation (r = 0.61), while the other comparisons all showed strong correlations. This indicates the HMM-SM may be more sensitive to changes in sensor configuration, whereas DTW and MDP are relatively invariant to sensor placement. Several factors could explain this. Firstly, the HMM training might be more inconsistent due to limitations in sample size or issues with hyperparameter selection. This inconsistency can affect the performance of HMM-SM independently of any changes in the sensor configuration. Another reason could be the fundamental differences between HMM-SM and the other methods. DTW and MDP are relatively simple distance measures that directly compare the time-series data. In contrast, HMM-SM involves a more complex analysis, as it compares the learned patterns from the sensor signals through a probabilistic model. This more abstract approach might make HMM-SM more sensitive to variations in sensor configuration. The increased consistency across the sensor configurations in the DTW and MDP algorithms could offer greater flexibility when integrating them into wearable systems. This flexibility might allow for sensor placement decisions based on other factors such as type of ambulatory analysis [51], accurate gait event detection [14], or activity analysis [52].

Correlations between the inertial sensor methods suggest some convergent validity among the different inertial sensor-based measures, indicating that various methods can extract similar relevant features from gait. For example, the HMM-SM lower leg results were significantly correlated with both the MDP pelvis configuration and the DTW lower leg and pelvis configurations. The DTW lower leg configuration showed significant correlation with the INI, suggesting that parameter-based and inertial signal-based approaches can provide similar evaluations of LLPU gait. This supports the idea that we can use inertial signals to implicitly assess changes in overall spatiotemporal and kinematic gait aspects such as those used by the INI and the GPS.

The DTW and MDP algorithms were highly correlated with each other. At a conceptual level, the MDP is similar to DTW in that for each point along the gait cycle, the MDP uses Euclidean distance to find the closest matching point in the SOM trained on reference gait. DTW employs an analogous strategy but includes additional constraints on the warping window (i.e., how far before or after to search for matching points) and the allowable warping path [53]. Although training a SOM can be computationally expensive [54], it needs to be trained only once on the reference dataset. Thus, the trade-off between computational complexity and performance should be evaluated when determining the most applicable method.

### Correlations with self-report measures

Neither the LCI-5 nor the PEQ-MS exhibited significant correlations with the gait quality measures including GPS. This could be due to the sample size, and larger sample sizes may be needed to reveal potential existing correlations. However, previous studies have underscored the potential differences between gait quality measures and measures of gait function or self-reported capacity [55, 56]. While some research has indicated positive correlations between improvements in gait quality measures (e.g., quantified gait parameters, summarized gait indices like the Gait Deviation Index) and functional assessments like the Functional Gait Assessment [57] or the Timed Up and Go test [55], others have found low correlates between gait measures and with functional measures like gait speed [56] or self-report measures of ambulation [55]. The results of this study further highlight the distinction between gait quality and ambulatory function or capacity, emphasizing the importance of developing methods that can monitor gait quality over the long term.

### Gait quality results across prosthetic levels

All measures (at various sensor locations) detected differences between at least one set of subgroups. The HMM-SM pelvis configuration and INI discriminated between the most sub-groups (n = 3). The rest of the measures including the GPS discriminated between one or two sub-groups. Additionally, if we consider only tracking a single sensor location (in cases where the goal is to maximize wearability of the gait assessment system), then the HMM-SM is the only measure to discriminate between 3 subgroups.

The variability in the discriminative performance among gait quality measures correlated with the GPS further underscores that while different inertial sensor-based measures can assess overall gait quality, each measure may have its own strengths and specific performance characteristics. For example, the HMM-SM may be particularly effective for monitoring pelvic kinematics (or other kinematic changes for individuals with lower-limb difference), whereas DTW could more closely approximate GPS performance.

Contrary to other studies comparing TT and TF gait [32, 58, 59], neither the GPS nor any of the signal-based measures (HMM-SM, MDP, and DTW) identified differences between the TT and TF groups. This might be attributed to the fact that 4 out of the 7 TF amputees used microprocessor knee joints, which significantly improve gait patterns, potentially approaching able-bodied gait kinematics [60].

The HMM-SM was particularly effective at discriminating the LS group. Individuals with limb discrepancy often use pelvic compensatory strategies and display increased pelvic obliquity (i.e., left or right side of the hip higher than the other) [61]. This may explain why the pelvis configuration consistently differentiated the LS group from the others, suggesting the HMM-SM may be more responsive to changes in pelvic kinematics than the GPS. It also could indicate that the HMM-SM is particularly effective at monitoring the compensatory strategies employed by individuals with limb discrepancy (pelvic or otherwise), as evidenced by the performance of both the lower leg and pelvis configurations.

### Future work and limitations

This study identified significant correlations between the GPS and the HMM-SM as well as between the GPS and other inertial sensor-based methods, supporting the use of inertial sensors for monitoring overall gait quality. Furthermore, the study involved a diverse LLPU group from 9 to 63 years of age, comprising various prosthetic types—transfemoral, transtibial, Van Nes, and congenital limb shortening—as well as a mix of unilateral and bilateral LLPU. This suggests that the HMM-SM and other inertial signal-based measures can be applied to assess a wide range of gait characteristics and deviations.

However, future research should seek to address several study limitations. The study protocol did not allow for repeatability testing, as we only collected gait data during a single session for each participant. Among the inertial sensor methods evaluated, only the MGS reported repeatability outcomes [15]. Reliability is crucial for gait metrics to detect significant changes in gait patterns [62], so future studies should include test–retest assessments over multiple time periods without intervention to evaluate the repeatability of inertial sensor-based gait quality measures. It should be highlighted that the inertial sensor methods were validated against GPS using kinematics from an inertial sensor system (the Xsens MVN) as opposed to kinematics from an optical/camera motion capture system. Although XSens MVN has been well-validated as described in the methodology, this could affect the results or be a source of differences if future studies were to use alternative methods (e.g., optical systems) for calculating the GPS.

Additionally, future research should attempt to involve larger sample sizes. While the sample size used for the LLPU in this study (n = 26) was comparable to those used in existing studies such as for the INI (n = 8) [10] and LLPU GPS validation (n = 20) [55], more comprehensive validation studies for the GGI and GPS involved 64 and 407 participants for the gait disability cohorts, respectively [7, 35]. Larger sample sizes would enhance our ability to assess the correlation between the HMM-SM and the GPS and determine if any non-linear correlations exist. It could also enable us to assess correlations among the specific LLPU subgroups, which could reveal additional relationships not seen in the study or provide further evidence for correlations between the inertial sensor measures and the GPS. Future work should also continue hyperparameter testing, particularly for the HMM-SM, to explore the effects on correlation with the GPS and whether the methods from this study are broadly applicable to larger data sets. The HMM-SM exhibited more variability in performance than DTW or MDP among the different sensor configurations, which could also be indicative of overfitting by the model during the HMM training. Future studies should assess variance in the hyperparameter learning between sets of similar gait patterns (e.g., trials/sets of 100 gait cycles where gait is expected to not have changed, verified by similar GPS scores) to evaluate whether HMM training is consistent. Potential addition of regularization to the HMM training step or hyperparameter tuning (such as number of HMM states) may be necessary in the case of inconsistent HMM training or overfitting.

Although this study included correlation results for the GPS compared to parameter-based inertial sensor methods (INI and MGS), it did not explore correlations between individual parameters and the GPS. This was primarily due to this work’s focus on exploring the validity of signal-based measures, which could reduce the need for more complex multi-sensor systems. Future studies with larger sample sizes could investigate correlations among gait parameters, including but not limited to those used in INI and MGS, to determine whether a minimal set of gait parameters and number of sensors could accurately estimate the GPS. Furthermore, it could elucidate gait parameters which strongly contribute to (or are affected by) overall gait quality and gait kinematics.

Long-term research should also test the HMM-SM and other methods in more natural, free-living conditions, such as in environments without laboratory assistance or during natural walking conditions like on flat sidewalks. Inertial sensor-based methods are intended for integration into systems that clinicians and individuals can use during routine activities. Therefore, it is important to examine whether variations in sensor placement and orientation or less controlled walking conditions affect their performance.

While able-bodied gait patterns were used in this study, as in other studies evaluating gait quality measures [7, 10, 35], future applications could also investigate using other reference sets such as high-functioning LLPU individuals to determine whether able-bodied gait is a relevant and attainable gold standard for LLPU or other disability populations. Finally, future studies should evaluate the validity of inertial sensor-based gait quality measures in other populations to ensure its generalizability and effectiveness across different disability groups.

## Conclusions

The HMM-SM, along with several other inertial sensor-based measures, demonstrated significant correlation with a clinically validated measure of gait quality when tested on a diverse group of prosthetic users. This preliminary evidence suggests that the HMM-SM could be an effective tool for assessing gait quality. Additionally, results from the HMM-SM, DTW, and MDP indicate that overall gait quality could be assessed using just the signals from a small set of inertial sensors. These methods could offer an easily interpretable assessment of gait pattern deviations without requiring extensive parameter tuning or model training, making them adaptable to a wide range of gait deviations.

Given the substantial time pressures faced by clinicians, it is crucial to develop systems that are quick and easy to use to encourage their adoption in clinical settings. Furthermore, inertial sensor-based methods can enable continuous gait monitoring outside the clinic and support the ongoing assessment of gait progress and long-term changes in gait patterns.

## Data Availability

No datasets were generated or analysed during the current study.

## Abbreviations

DTW: Dynamic Time Warping
GD: Gait duration
GGI: Gillette Gait Index
GPS: Gait Profile Score
HMM: Hidden Markov Model
HMM-SM: Hidden Markov Model-based Similarity Measure
INI: Inertial Measurement Unit-based Gait Normalcy Index
LCI-5: Locomotor Capabilities Index-5
LLPU: Lower-Limb Prosthetic User
LS: Individual with Limb Shortening
MAB: Maximum ankle abduction
MAD: Maximum ankle adduction
MDP: Movement Deviation Profile
MGS: Multifeature Gait Score
MH: Maximum ankle height
MHD: Ankle horizontal displacement at MH
MV: Maximum ankle velocity
PEQ-MS: Prosthetic Evaluation Questionnaire-Mobility Section
PSP: Percentage swing phase
PCA: Principal Component Analysis
RM-ANOVA: Repeated Measures Analysis of Variance
SL: Stride length
SRM: Shank range of motion in swing phase
TF: Transfemoral amputee
TT: Transtibial amputee
VN: Van Nes amputee
